# The Effect of Eight Weeks of Passive Heat Therapy on Mental Health, Sleep, and Chronic Pain in Persons with Spinal Cord Injury: A Pilot Study

**DOI:** 10.3390/jcm14103566

**Published:** 2025-05-20

**Authors:** Hannah Uhlig-Reche, Sven Hoekstra, Yubo Wu, Dean Lundt Kellogg, Terry Romo, Christof A. Leicht, Michelle B. Trbovich

**Affiliations:** 1 Department of Rehabilitation Medicine, University of Texas Health at San Antonio, San Antonio, TX 78229, USA; kelloggd@uthscsa.edu (D.L.K.J.); trbovichm@uthscsa.edu (M.B.T.); 2 South Texas Veteran’s Health Care System, San Antonio, TX 78229, USA; wuy3@uthscsa.edu; 3 Department of Exercise and Sport Science, St. Mary’s University, San Antonio, TX 78228, USA; shoekstra@stmarytx.edu; 4 The Peter Harrison Centre for Disability Sport, School of Sport, Exercise and Health Sciences, Loughborough University, Loughborough LE11 3TU, UK; 5 Department of Medicine, University of Texas Health at San Antonio, San Antonio, TX 78229, USA; c.a.leicht@lboro.ac.uk

**Keywords:** spinal cord injury, passive whole-body heating, thermal therapy, mental health, sleepiness, chronic pain

## Abstract

**Background**/**Objectives**: Rates of depression, anxiety, sleep disturbances, and chronic pain are higher in people with spinal cord injury (SCI) compared with able-bodied individuals. Passive heat therapy (PHT), which raises core body temperature, may be an accessible therapeutic intervention. The effects of PHT on mental health, sleep, and pain in persons with SCI are unknown. **Methods**: We performed a time-controlled pre–post intervention pilot study in which ten veterans with chronic SCI underwent an 8-week supervised PHT intervention to raise oral temperature by 1 °C each session. Outcome measures were the 5-item Mental Health Inventory, Epworth Sleepiness Scale, and International Spinal Cord Injury Pain Extended Data Sets version 1.0. **Results**: There were no adverse events related to the intervention and nine out of ten participants completed all their intervention sessions. There was a reduction in pain intensity (*p* = 0.039) upon completing the intervention (from a median (IQR) of 2.0 (0.0, 3.5) to 1.0 (0.0, 4.5) on a 0–10 scale). However, there were no improvements in self-reported mental health or sleep outcomes (*p* > 0.339). **Conclusions**: This pilot study suggests that supervised repeated passive heat therapy may confer benefits for chronic pain in veterans with chronic SCI. Follow-up studies with larger sample sizes and more extensive sets of chronic pain outcomes are needed to confirm these findings.

## 1. Introduction

Rates of depression, anxiety, sleep disturbances, and chronic pain are higher in people with spinal cord injury (SCI) compared with able-bodied individuals [1,2,3,4,5]. For example, the prevalence of depression in the SCI population is estimated to be as high as 60%, while a meta-analysis of multi-national studies found that the estimated prevalence of anxiety in people with SCI ranges from 15 to 32% [2,6]. Furthermore, sleep disturbances are highly prevalent in persons with SCI [7]. These may include poor sleep quality, circadian rhythm sleep–wake disorders, insomnia, sleep-disordered breathing, restless leg syndrome, and periodic limb movement disorder [1,8]. Finally, the prevalence of chronic pain in persons living with SCI is estimated at ~68%, compared with ~17% in the general population [3,9].

Several non-pharmacological interventions to improve mental-health-related outcomes, sleep, and chronic pain have been explored in persons with SCI [10,11,12,13,14]. For example, a meta-analysis found that exercise can improve overall well-being and psychological well-being in persons with SCI [15]. Mindfulness exercises lead to reduced signs of depression and improve pain management [16]. Moreover, thermal interventions for improvements in mood and well-being have been practiced for centuries; however, their scientific underpinning remains limited when applied to individuals with SCI. Autonomic dysfunction in persons with SCI results in the inability to redistribute blood volume appropriately in response to heat, while sweating capacity is also abolished below the lesion level; thus, the therapeutic effects of heat exposure seen in able-bodied individuals may differ in persons with SCI [17]. Examples of thermal modalities include passive heat therapy (PHT) through sauna bathing, hot water immersion, and heating blankets. These have the advantage that they are accessible for persons at the lowest end of the physical capacity spectrum and, if proven effective, may be a health intervention applicable to most individuals with SCI.

There is preliminary evidence that PHT reduces pain outcomes in clinical populations other than individuals with SCI, particularly as it relates to musculoskeletal pain [18]. For example, 2 to 3 weeks of sauna bathing led to substantial reductions in perceived pain in individuals with fibromyalgia [19]. These findings may have been related to a desensitization of the nerve endings and, consequently, an increase in the pain threshold as observed following acute heat exposure in persons with rheumatoid arthritis [20]. Another potential reason for the improved pain perception includes a reduction in pro-inflammatory marker concentration following PHT [21,22]. At the same time, signs of depression were also reduced following 3 weeks of sauna bathing in persons with fibromyalgia. A meta-analysis of hydro- and balneotherapy interventions confirmed these findings by reporting improved anxiety and depression in healthy individuals and those with a chronic health condition [19]. This effect may be indirect via the reduction in pain but may also be explained by a reduction in inflammation or an elevation in the concentration of plasma β-endorphin as observed following acute heat exposure [23]. Similarly, the improvements in self-reported sleep outcomes following PHT may be related to reductions in pain, stress, and anxiety but also to alterations in the circadian variation in core body temperature in response to heat exposure, particularly before bedtime [24,25,26,27,28].

While the above studies underscore the potential efficacy of PHT to improve sleep, pain, and mental-health-related outcomes, there is a paucity of studies investigating this intervention in persons with SCI. The research conducted to date on PHT in SCI is mostly related to the investigation of acute responses to a single heat exposure and outcomes related to cardiometabolic health and autonomic function [29,30,31,32]. Nonetheless, these studies suggest that PHT is safe and tolerable to use by persons with SCI and does provide a sufficient stimulus to alter physiological outcomes. It has yet to be confirmed whether these changes translate to improvements in outcomes related to sleep, mental health, and chronic pain. Therefore, this pilot study investigates the effect of repeated PHT on mental health, sleep, and chronic pain in individuals with SCI. It is hypothesized that eight weeks of repeated PHT improves self-reported outcomes of mental health, sleep, and chronic pain when compared to a control time period.

## 2. Materials and Methods

### 2.1. Participants

Participants were adults with chronic SCI (>1 year). Participants were all veterans and recruited from the outpatient SCI clinic of the Audie L. Murphy Memorial Veterans Hospital in San Antonio by a spinal cord injury physician face-to-face at a regular clinic visit. Exclusion criteria included currently smoking, daily administration of anti-inflammatory or vasoactive medications, current pressure ulcer or skin breakdown, a history of heat-related illness, any active acute illness, and a baseline hemoglobin concentration of less than 11 g/dL documented within six months of study initiation. After providing information about this study’s procedures—approved by the University of Texas Health Science Center at San Antonio Institutional Review Board—all participants provided written informed consent.

### 2.2. Study Design and Procedures

In this pre–post intervention pilot study, participants served as their own control with questionnaires administered on study visit 1 (baseline), after eight weeks without intervention (control), and 2–4 days following an eight-week PHT protocol (intervention). All procedures took place in the Audie L. Murphy Memorial Veterans Hospital in San Antonio. Participants filled out the questionnaires in a quiet space, whilst resting on a hospital bed or in their personal wheelchair. During the PHT intervention period, participants visited the laboratory thrice weekly. Prior to each session, baseline oral temperature was obtained by sublingual placement of a temperature probe for 5 min. Thereafter, a water-perfused suit with the infused water set at 48 °C was placed over the torso, while three heated fleece-lined electrical blankets set at 43 °C and an aluminum foil blanket were placed over the entire body. Each session was completed when oral temperature was elevated by 1 °C from baseline, or when the participant requested to terminate due to thermal discomfort. Study enrollment and intervention occurred between January 2023 and July 2024.

### 2.3. Outcomes

Validated surveys were utilized as follows: the 5-item Mental Health Inventory (MHI-5), Epworth Sleepiness Scale (ESS), and the International Spinal Cord Injury Pain Extended Data Sets version 1.0 (ISCIPEDS).

The MHI-5 has shown good reliability and validity in screening for general mental health issues in individuals with functional impairments [33,34]. This 5-item instrument assesses the frequency of various emotional states with a 0–6 score per item. Responses were scored and transformed to a 0–100 scale as performed elsewhere [35]. Scores were dichotomized based on ≤56 being indicative of general mental health problems and >56 suggesting no general mental health problems [35].

The ESS is a 7-item questionnaire that measures daytime sleepiness in various situations using a 0–3 score per item [36]. A score of greater than 10 is indicative of excessive daytime sleepiness. Scores were dichotomized into normal (scores 0–10) or excessively sleepy (>10) for further analysis.

The ISCIPEDS comprises seven questions assessing pain frequency, highest pain intensity, average unpleasantness of pain, frequency of tolerable pain, current pain intensity, pain duration, and timing of pain intensity [37]. Higher scores indicate more severe and/or frequent pain.

### 2.4. Statistical Analysis

Participant characteristics are shown as mean ± SD. Counts and percentages were calculated for categorical data. The questionnaire data for each of the three assessment time points (i.e., baseline, control, and intervention) were compared using Friedman tests. Post hoc pairwise comparisons were performed using Wilcoxon’s Signed Rank tests. The Chi-square test was used to assess differences in observed frequencies. All available participant data were included as an intention-to-treat analysis. Cohen’s d effect sizes (ES) and the 98% confidence interval around the median difference for control versus intervention were calculated for each outcome measure, with the magnitude of the ES classed as small (0.20), moderate (0.50), and large (0.80). A probability of <0.05 was used as the threshold to accept statistical significance. All statistical tests were conducted using the 28th version of SPSS.

## 3. Results

Fifteen veterans were invited to participate, of which two female and eight male veterans with chronic SCI were enrolled in this study (Table 1). Nine participants completed all prescribed passive heating sessions, while one withdrew after 18 sessions due to an adverse event unrelated to the intervention; post-intervention questionnaires are unavailable for this participant. The intervention was well-tolerated and there were no related adverse events, thus clearly demonstrating the safety and tolerability of PHT in the SCI population.

### 3.1. Mental Health (Figure 1)

**Figure 1:**
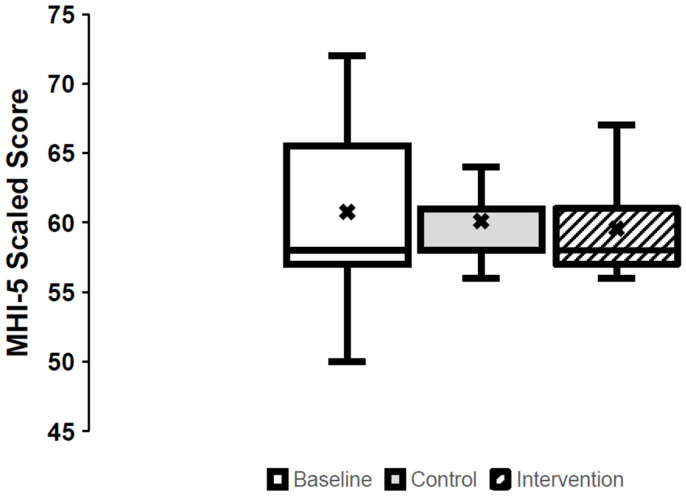
The scaled Mental Health Inventory-5 scores at baseline, after the control period (control), and after the passive-heat-therapy intervention (intervention). Box plots represent median, quartiles, and minimum and maximum, while crosses represent means.

At baseline, two participants screened positive for general mental health problems based on the MHI-5, which remained consistent at control and intervention. The scaled MHI-5 score at baseline was 60.8 (6.2), which did not change following the control or intervention period (*p* = 0.867; ES: 0.06; CI: −2.8–0) (Figure 1).

### 3.2. Sleepiness

One participant screened positive for excessive daytime sleepiness at baseline. This progressed to three and two participants after the control and intervention, respectively. At the group level, ESS was 7.9 (5.9) at baseline, and did not change after the control (7.3 (4.6)) or intervention (8.7 (5.0)) (*p* = 0.339; ES: 0.29; CI: −2–4).

### 3.3. Pain (Table 2)

At baseline, five participants reported experiencing pain every day of the week. This was also true at control but was reduced to four participants at intervention. The median number of days during which participants experienced pain did not change between the three time points (*p* = 0.156). The median reported pain intensity was three out of ten at baseline, which reduced to one out of ten following the intervention (*p* = 0.039). The distribution of the reported pain duration differed between the assessment periods (*p* = 0.008), with a reduction in the number of participants reporting pain lasting more than 24 h (Table 2).

**Table 2:** Pain assessment for the last 7 days based on the International Spinal Cord Injury Pain Extended Data Set (version 1.0).

Question	Baseline	Control	Intervention	*p*-Value	ES (CI)
Number of days with pain, median (IQR)	7.0 (0.0, 7.0)	6.5 (2.0, 7.0)	4.0 (0.0, 7.0)	0.156	0.25 (−2–0)
Worst pain intensity in last week, median (IQR)	3.0 (0.50, 7.50)	3.0 (0.0, 5.3)	3.0 (0.0, 6.0)	0.076	0.16 (0–1)
Pain unpleasantness, median (IQR)	2.0 (0.5, 6.0)	3.0 (0.0, 4.3)	2.0 (0.0, 3.0)	0.504	0.29 (−2–0)
Number of days with manageable/tolerable pain, median (IQR)	5.5 (1.0, 7.0)	5.0 (0.0, 7.0)	3.0 (0.0, 6.0)	0.494	0.28 (−4–0)
Pain intensity presently, median (IQR)	3.0 (0.0, 5.5)	2.0 (0.0, 3.5)	1.0 (0.0, 4.5)	**0.039**	0.29 (−3–0)
Length of time the pain lasts for, *n* (% of respondents)				**0.008**	
≤1 min	1 (10)	2 (20)	2 (20)		
>1 min but <1 h	0 (0)	0 (0)	0 (0)		
≥1 h but <24 h	2 (20)	1 (10)	1 (10)		
≥24 h	1 (10)	1 (10)	0 (0)		
Constant/continuous	4 (40)	5 (50)	5 (50)		
Unknown	1 (10)	1 (10)	1 (10)		
Unanswered	1 (10)	0 (0)	1 (10)		
When during the day is the pain most intense, *n* (% of respondents)				0.174	
Morning (06:01–12:00)	1 (10)	1 (10)	1 (10)		
Afternoon (12:01–18:00)	1 (10)	0 (0)	0 (0)		
Evening (18:01–24:00)	2 (20)	1 (10)	3 (30)		
Night (0:01–06:00)	1 (10)	4 (40)	3 (30)		
Unpredictable (pain is not consistently more intense at any one time of day)	3 (30)	4 (40)	2 (20)		
Unanswered	2 (20)	0 (0)	1 (10)		

Abbreviations: IQR = interquartile range; ES: effect size; CI: confidence interval.

## 4. Discussion

In this pre–post PHT intervention pilot study with a time-control phase, we found improvements in self-reported pain measures but no changes in self-reported mental health or sleepiness. Together with the excellent intervention adherence—with no intervention-related drop-out—this may provide impetus to further explore the use of PHT in SCI chronic pain management. To our knowledge, there are no other published studies investigating the effect of PHT on mental health, sleep, or chronic pain in persons with SCI. As such, the effects of PHT interventions in other populations are discussed to contextualize our findings.

Following the eight-week intervention phase, we observed improvements in some aspects of the ISCIPEDS, the international chronic pain questionnaire developed for persons with SCI [37]. Specifically, we found a 1-point reduction in median pain intensity, combined with a small effect size. While there is no consensus on what constitutes a clinically meaningful change in aspects of the ISCIPEDS specifically, the literature investigating the able-bodied population suggests that a 2-point reduction on a 0–10 scale can be considered clinically meaningful [38]. It should be noted, however, that the baseline pain intensity was already relatively low (2 on a 0–10 scale). A floor effect may therefore have occurred. There was also a statistically significant reduction in the number of participants reporting pain duration longer than 24 h—this is attributable to one participant’s experience in our relatively small sample.

The reduction in pain found in the current study was similar compared to several other studies, although larger effects have been reported too [39,40,41,42]. Meta-analyses of randomized controlled trials in persons with fibromyalgia and chronic lower back pain indicate a PHT-induced pain reduction between 0.42 and 1.61 on a 0–10 scale compared to the control groups [39,43]. This suggests that our finding in persons with SCI is in line with other populations. On the other hand, dry sauna therapy performed twice daily for five consecutive days led to a reduction from 5 to 3 on a 0–10 scale in participants with chronic low back pain. In addition, Chadwick et al. (2025) [44] reported a reduction in pain sensation from 5.4 to 3.8 following four weeks of hot water immersion, while the fibromyalgia patients investigated by Donmez et al. (2005) [45] reduced their pain scores from 6.8 to 2.9 after 2 weeks of sauna bathing. Aside from the higher baseline pain scores observed in these participants, the heating protocols may also partly explain the larger observed effect in these studies. For example, Donmez et al. and Cho et al. employed daily or even twice-daily sauna sessions, while Chadwick et al. increased core temperature to 38.5 °C compared to the 1 °C rise in the current study [42,44,45]. Thus, while we found a statistically significant reduction in pain intensity, future studies may employ more frequent and/or more intense heating protocols to further enhance its efficacy.

There are several potential reasons for the analgesic effect of PHT. First, pain and heat share a sensory receptor, the transient receptor potential vanilloid 1 (TRPV1). By repeatedly heating the core and skin through PHT, TRPV1 receptors may be desensitized, leading to a lower sensitivity to painful stimuli [43]. In addition, there is increasing evidence for the role of inflammation in chronic SCI pain, particularly below the lesion [46]. PHT can lead to a reduction in the concentration of plasma pro-inflammatory cytokines, providing an additional mechanistic possibility for the observed effect [47,48]. Taken together, despite the modest effect of the pain-related findings, they provide impetus for follow-up studies into the therapeutic role of PHT in pain management. Of note, PHT did not have the sedating side effects that pharmacologic pain treatments often have, as indicated by the lack of change in sleepiness scores.

We found no improvements in mental health following the PHT intervention. This tends to be in contrast with studies on PHT and mental health in able-bodied individuals, with and without depression [46,49,50]. In a cross-sectional study on a sample of 1180 Swedish adults, frequency of sauna bathing was positively associated with ratings of happiness and mood [24]. Furthermore, a study comparing a single session of whole-body hyperthermia to a sham intervention in depressed adults resulted in a significant anti-depressant effect that persisted for six weeks, while 4 weeks of sauna bathing in persons with mild depression also improved mental and somatic complaints [49,51]. Importantly, a meta-analysis by Hanusch and Janssen indicated that there is a linear relationship between the core temperature attained during heating and the intervention’s effect on mental health [50]. Based on this analysis, the authors suggest heating protocols that reach a core temperature of 38.5 °C or higher in order to improve mental health outcomes. Whilst this analysis was based on studies investigating people with depression—who have more negative baseline scores than the current participants—it does suggest that the PHT protocol used in the present study was not sufficiently intense to improve mental health. Finally, it should be noted that there was large variability in the MHI-5 outcomes, suggesting insufficient power in this pilot study and underscoring the need for larger-scale follow-up studies.

Similarly, there was no intervention effect on sleep variables measured by the ESS. Although research is limited on the effect of PHT on sleep in SCI, PHT in able-bodied individuals has been associated with reduced sleep onset latency, increased deep sleep, enhanced sleep efficiency, and improved overall sleep quality [27,28,52,53]. Sleep benefits in able-bodied elderly individuals have been noted when a warm bath was taken in the evening time, though the PHT in the current study was performed during the early daytime hours [28]. It is important to note that the ESS is limited by it being a representation of the perception of daytime sleepiness rather than a direct measurement of sleep quality itself. Future studies investigating PHT in SCI should, therefore, consider assessing sleep quality by wrist-worn actigraphy or polysomnography, as well as including questionnaires that more specifically target sleep quality (e.g., the Pittsburgh Sleep Quality Index) [54]. Taken together, PHT had little effect on mental health and sleep in our cohort. While the large variability in the measurements may be a factor, the modest intensity of the heating protocol may also partly explain this finding. An additional proposed mechanism for the benefits of PHT for sleep and mental health is through an increased synthesis and release of neurotrophins and neurotransmitters [55]. Using a similar heating mode, we previously showed that modest heating does not lead to an acute increase in brain-derived neurotropic factor concentration as compared to traditional whole-body heating, suggesting that more intense heating may be needed to modulate these neuronal processes [56].

The strengths of this study include the novelty of investigating the effect of PHT on mental health, sleep, and pain outcomes in persons with chronic SCI. There was a low attrition rate with the only drop-out occurring due to a matter unrelated to this study. The intervention was well-tolerated in the supervised setting. On the other hand, this study also had several limitations. Aside from the small sample size, the generalizability of the results is limited by the inclusion of veterans only. Veterans likely have different experiences with mental health, sleep, and pain compared to non-veterans [57]. Follow-up studies should include a control group, preferably one that undergoes a sham-control intervention. Lastly, we did not account for medication use during the study period, such as those that may impact mental health, sleep, or pain.

In conclusion, supervised PHT may have the potential to serve as a safe, tolerable, and feasible adjunct to pain management in persons with chronic SCI. However, its effects on sleep and mental health may be limited. Further investigation with a larger sample size and a more extensive set of chronic pain outcomes is currently being planned to take the next steps toward the clinical implementation of this intervention.

## Figures and Tables

**Table 1:** Participant characteristics.

Age in years, mean (SD)	45 (14)
Years since injury, mean (SD)	9.2 (10.5)
Lesion level at time of study, *n* (%)	
Cervical, C4–C7	4 (40)
Thoracic, T5–T12	6 (60)
AIS completeness at time of study, *n* (%)	
A	4 (40)
B	1 (10)
C	4 (40)
D	1 (10)
BMI in kg/m^2^, mean (SD)	28.0 (3.0)

Note: AIS = American Spinal Injury Association Impairment Scale; SD = standard deviation; *n* = count.

## Data Availability

Data are available upon request from the corresponding author.
